# The Development and Effect of Systemic Hypertension on Clinical and Radiological Outcome in Adult Moyamoya Angiopathy Following Revascularization Surgery: Experience of a Single European Institution

**DOI:** 10.3390/jcm12134219

**Published:** 2023-06-23

**Authors:** Kristin Lucia, Güliz Acker, Kerstin Rubarth, Defne Beyaztas, Peter Vajkoczy

**Affiliations:** 1 Department of Neurosurgery, Charité—Universitätsmedizin Berlin, Corporate Member of Freie Universität Berlin and Humboldt-Universität Zu Berlin, Charitéplatz 1, 10117 Berlin, Germany; kristin.lucia@kgu.de (K.L.); gueliz.acker@charite.de (G.A.); defne.beyaztas@charite.de (D.B.); 2 Department of Neurosurgery, Goethe University Hospital Frankfurt Am Main, Schleusenweg 2-16, 60528 Frankfurt Am Main, Germany; 3 Berlin Institute of Health, Charité—Universitätsmedizin Berlin, Charitéplatz 1, 10117 Berlin, Germany; kerstin.rubarth@charite.de; 4 Institute of Biometrics and Clinical Epidemiology Universitätsmedizin Berlin, Charitéplatz 1, 10117 Berlin, Germany; 5 Institute of Medical Informatics Universitätsmedizin Berlin, Charitéplatz 1, 10117 Berlin, Germany

**Keywords:** systemic hypertension, moyamoya angiopathy, autoregulation, cerebral perfusion

## Abstract

Patients with Moyamoya Angiopathy (MMA) display structurally altered vessels with decreased cerebral autoregulatory capacity, so aggressive lowering of systemic hypertension may aggravate ischemic symptoms, whereas uncontrolled hypertension may promote hemorrhage. This study provides an in-depth analysis of the role of hypertension in adult MMA patients including long-term analysis of clinical and radiological development. In this single-center retrospective analysis of 137 adult MMA patients with 206 surgically treated hemispheres angiographic images, clinical/operative data were reviewed and scored. Univariate Cox-regression analysis was performed to evaluate hypertension as a predictor for negative angiographic and clinical outcomes following revascularization surgery. A total of 50% of patients were being treated for hypertension prior to the first surgery. Patients with and without hypertension did not differ in terms of age, gender, diagnosis, symptom onset or disease severity (Berlin and Suzuki Grades). Although hypertension did not statistically significantly affect postoperative collaterals, moyamoya vessels or STA-MCA bypass patency, patients with hypertension showed higher rates of bypass patency and better bypass filling compared to those without hypertension. No significant differences in adverse events were found in patients with and without systemic hypertension and the presence of systemic hypertension was not found to predict negative clinical or radiological outcomes. In conclusion, the rate of systemic hypertension in MMA patients appears to be higher than the general population; however, this is not associated with an increased risk of postoperative complications or negative angiographic development following revascularization procedures. Systemic hypertension may also positively influence the rate of bypass patency and filling following revascularization procedures.

## 1. Introduction

Under physiological conditions, cerebral perfusion is subject to autoregulatory mechanisms which maintain cerebral blood flow changes in systemic blood pressure [1]. This is accomplished by the integration of metabolic, myogenic and neurogenic control mechanisms within the cerebral vasculature [2]. This regulatory system, however, can only be maintained within a certain range of systemic blood pressure (generally mean arterial pressure between 50–150 mmHg) before compensatory mechanisms are exhausted and cerebral blood flow passively adapts to systemic pressure resulting in either ischemia or hemorrhage [2].

In patients with Moyamoya angiopathy (MMA), a rare stenoocclusive cerebrovascular disease affecting the basal cerebral arteries, fragile net-like vessels with loss of autoregulatory mechanisms are formed [3]. As such, patients with MMA can present with intracranial hemorrhage from abnormal vascular structures as well as ischemic events [4,5]. To date, surgical revascularization remains the primary treatment for MMA as it aims at restoring perfusion to stabilize cerebrovascular hemodynamics and reduce hemorrhagic and ischemic events [6,7]. Although specific recommendations for blood pressure targets in MMA are lacking, the role of arterial blood pressure has more recently emerged as an important aspect of long-term disease management in patients with MMA [8,9,10]. Patients with MMA have recently been found to have a higher risk of suffering from systemic hypertension than general controls [11]. Asian cohorts of pediatric MMA patients have been found to present with a high prevalence of essential hypertension (29%) [8], and in a series of 200 North American adult patients, this increased up to 53% [12]. Longitudinal data on the possible effects of systemic hypertension on angioarchitecture or the occurrence of clinically relevant adverse events in adult MMA patients, however, have not yet been generated. Due to the structurally altered moyamoya vessels with decreased autoregulatory capacity to adapt to systemic blood pressure, aggressive lowering of systemic hypertension may therefore aggravate ischemic symptoms, whereas uncontrolled hypertension may promote hemorrhage and chronic hypertension per se is associated with diverse cardiovascular and neurological sequelae [13].

While the precise pathomechanism of systemic hypertension in MMA remains unclear, it has been proposed that it is a compensatory response resulting from chronic decreased cerebral perfusion due to structural abnormalities of moyamoya vessels and impaired vasomotor response [14]. Secondary hypertension as a result of vascular stenosis elsewhere (for example renal artery stenosis) or endocrine disease may also be present in MMA patients. As such, traditional antihypertensive medication may not be effective in these patients. It has therefore been proposed that surgical revascularization performed by extra-intracranial bypass may be seen as beneficial for the treatment of systemic hypertension regarding cerebral autoregulation as well as the systemic sequelae resulting from it [8]. The aim of the current study is therefore to characterize the incidence of systemic hypertension in adult MMA patients in our institution, and whether revascularization surgery affects the incidence of hypertension. To this end, we examined the presence of systemic hypertension based on the presence of pharmacological treatment before and one year after surgical intervention. We then assess whether patients with and without preoperative systemic hypertension differ in regards to angioarchitecture development (bypass patency, leptomeningeal and intra-/extracranial collateralization as well as moyamoya vessels) following revascularization surgery. For this purpose, angiographic studies performed at the last available follow-up were analyzed. Finally, we analyzed whether or not the presence of arterial hypertension in MMA patients is associated with adverse clinical outcomes.

## 2. Materials and Methods

### 2.1. Study Design

The study was conducted as a retrospective analysis based on the data from medical records of adult patients undergoing surgical treatment for Moyamoya angiopathy (ICD code 167.5) in our department between 2007 and December 2020. Moyamoya Angiopathy is used to describe all forms of the disease, including Moyamoya disease (MMD), Moyamoya syndrome (MMS) and unilateral Moyamoya disease or syndrome (uMMD/uMMS). Definitions were used based on the guidelines established by the Research Committee for Moyamoya Disease and patients with possible associated diseases were classified as Moyamoya syndrome [4]. Patients were excluded from analysis if a concurrent disease causing secondary systemic hypertension was present. These include diagnosis of renal artery stenosis, endocrine disorders (adrenal gland tumors, thyroid disorders), aortic valve disease, aortic coarctation or polycystic kidney disease.

### 2.2. Variables

Electronic medical records were used to retrieve data on patient demographics, time of surgery, follow-up time, angiographic follow-up and history of complications, as well as MRI and hemodynamic diagnostics (obtained either by xenon-computerized tomography or acetazolamide-stimulated single-photon emission computed tomography). Data were included in the one-year follow-up if available from 11–13 months following surgery.

The severity of MMA was classified using the Suzuki [3] and Berlin Grading Systems [15]. Preoperative cerebrovascular reserve capacity (CVRC) was included in the calculation of the Berlin Grading. The qualitative assessment of the CVRC was performed as described before [16]. Briefly, CVRC was assessed qualitatively by visual interpretation with a standardized side-by-side display of anatomically normalized resting and acetazolamide images together with the rCVRC map. Impaired CVRC was defined as a missing signal increase after acetazolamide stimulation. If stimulation with acetazolamide led to an increase in the signal, CVRC was considered sustained.

The presence of clinically relevant systemic hypertension was determined based on whether patients were receiving pharmacological treatment with antihypertensive medications at the time points analyzed.

Surgical strategies employed in the revascularization of MMA patients included combined superficial temporal artery to middle cerebral artery (STA-MCA) bypass with encephalodurosynangiosis (EDS) and/or encephalomyosynangiosis (EMS), direct external carotid artery to middle cerebral artery (ECA-MCA) bypass or STA-MCA bypass alone. One case of indirect revascularization using encephalodurogaleosynangiosis (EDGS) was also performed.

### 2.3. Angiographic Analysis

Several aspects of MMA disease progression and revascularization results were analyzed based on angiographic exams performed pre-and postoperatively. These include the extent of STA-MCA bypass filling, bypass patency, leptomeningeal collateralization, intra-extracranial collateralization and presence of moyamoya vessels.

In particular, the grading of moyamoya vessel development was performed as previously described [17]. Bypass filling was determined as follows: grade 1 refers to the antegrade filing of one or two cortical MCA branches; grade 2 refers to ante- and retrograde filling of the MCA territory; grade 3 refers to filling of the entire MCA vascular territory [18]. Leptomeningeal collateralization was determined based on the presence or absence of anastomoses in the watershed zones of the posterior cerebral artery to the anterior and/or to the middle cerebral artery [19]. Intra-extracranial collateralization was examined based on the presence and degree (none, moderate, extensive) of transdural collaterals arising from the middle meningeal artery [20,21]. Development of moyamoya vessels was classified in orientation the previous study by Zhao et al. [22] as either absent, faint and localized or abundant. Bypass patency was divided into two categories, either patent (grade 1) or stenosed/occluded (grade 2).

Angiographic analysis was performed preoperatively and at last follow-up after revascularization surgery for each hemisphere individually. Results for the development of leptomeningeal and extra-intracranial collateralization are presented as the development observed at the last follow-up compared to the first described findings. Bypass patency and bypass filling are reported as the status at the last follow-up.

### 2.4. Statistical Analysis

Data are presented as absolute counts and percentages of total or median and range (as indicated). Group comparisons were performed by using the chi-square test for categorical variables and the Mann–Whitney U-test for metric variables. Paired data were analyzed using the McNemar test. Univariate Cox regression analyses were performed using the presence of arterial hypertension as a predictor of event occurrence in angiographic analysis. Due to the exploratory nature of this study, arterial hypertension was used as the sole predictor for event occurrence. Events included poor STA-MCA bypass filling (grade 1), the presence of watershed zone anastomoses for leptomeningeal collateralization, extensive transdural collaterals from the middle meningeal artery and abundant moyamoya vessels compared to the preoperative angiography. Bypass occlusion or stenosis as well as poor STA-MCA bypass filling were defined compared to the first postoperative angiography. Only patients with data available for a minimum of one year following the first surgery were included in the regression analysis. As no variables were found to approach the cut-off of *p* > 0.150, multivariate analysis was not performed.

All *p*-values of less than 0.05 were considered statistically significant; however, due to the exploratory nature of this setting, no adjustment for multiplicity was conducted and hence, *p*-values are interpreted in a hypothesis-generating way. All analyses were performed in SPSS (version 24; IBM Corp., Armonk, NY, USA).

## 3. Results

### 3.1. Patient Characteristics

We analyzed a total of 137 patients with 206 surgically treated hemispheres. All patients were operated in a single institution between 2007 and 2020 by the senior author. Of these, 68 (50%) patients (104 hemispheres) received treatment for preoperative hypertension prior to their first surgery, whereas 69 patients (50% and 102 hemispheres) were not being treated for hypertension prior to their first surgery (Table 1). We first examined baseline characteristics among patients with and without systemic hypertension prior to revascularization surgery.

#### 3.1.1. Demographic Data

The median age of patients with preoperative systemic hypertension was not significantly higher than those without preoperative systemic hypertension (44 versus 36 years, *p* = 0.147). Regarding gender distribution, both groups consisted primarily of female patients (64% of those with hypertension, and 80% of those without hypertension, *p* = 0.425). Our cohort consisted of mostly Caucasian patients (94% in both groups), followed by Asian (4% of patients with hypertension and 2% of patients without hypertension) and Middle Eastern (2% of patients with hypertension and 4% of patients without hypertension).

#### 3.1.2. Diagnosis and Symptom Onset

Moyamoya disease was the most common diagnosis in both patients with and without preoperative hypertension (followed by moyamoya syndrome and unilateral moyamoya disease) (Table 1). Ischemic symptoms were the most common form of disease onset in both patients with preoperative systemic hypertension and those without (Table 1).

In total, 137 patients (206 hemispheres) were analyzed. Data are presented as both the sum and percentage of total patients and or hemispheres (as indicated in each category) for patients with and without preoperative systemic hypertension. Suzuki scores were not available for 11 hemispheres in the group with preoperative hypertension and in 4 hemispheres in the group without preoperative hypertension. Berlin scores were not available for 14 hemispheres in the group with preoperative hypertension and in 8 hemispheres in the group without preoperative hypertension. In this case, percentages are given as the total of available hemispheres in each group, respectively. The surgical strategy includes both first and second surgeries in patients with bilateral disease and only one surgery for those with unilateral disease. Group comparisons were performed using the chi-square test for categorical variables and Mann–Whitney U Test for metric variables with *p* < 0.05 considered statistically significant.

### 3.2. Disease Severity

Preoperative MRI scans were available for 90 patients with systemic hypertension and 94 without systemic hypertension. Among these patients, ischemic lesions in 65 hemispheres (72%) of patients with systemic hypertension and in 67 hemispheres (71%) of patients without hypertension. Impaired CVRC was found preoperatively in 63 hemispheres (70%) among patients with preoperative systemic hypertension and 73 hemispheres (78%) in patients without preoperative systemic hypertension. Berlin scores were not available for 14 hemispheres (13%) in the group with preoperative hypertension and in 8 hemispheres (8%) in the group without preoperative hypertension either due to missing MRI scans or preoperative CVRC measurements. Disease severity as determined by the Berlin Grade was also similarly distributed in patients with and without preoperative systemic hypertension (*p* = 0.451) (Table 1).

Suzuki scores were not available for 11 hemispheres (11%) in the group with preoperative hypertension and in 4 (4%) hemispheres in the group without preoperative hypertension. As with the Berlin Grading, we also found no significant difference in the distribution of preoperative Suzuki scores between patients with and without preoperative systemic hypertension (*p* = 0.675) (Table 1).

Overall, we found no statistically significant differences in any baseline characteristics between patients with and without systemic hypertension prior to revascularization surgery.

### 3.3. Surgical Treatment

The most common treatment approach (by hemisphere) was via STA-MCA bypass and EDS followed by STA-MCA bypass alone. Further, less commonly implemented strategies included STA-MCA bypass plus EMS, EDGS and ECA-MCA bypass with RAG (Table 1).

### 3.4. Influence of Revascularization Surgery on the Incidence of Systemic Hypertension at One Year Following Intervention

To evaluate whether revascularization surgery affects the presence of systemic hypertension, only patients for which data were available at both time points preoperatively and at one year postoperatively were included, resulting in 39 patients with single hemisphere surgery and 29 with revascularization on both hemispheres. In the case of patients undergoing surgery of two hemispheres, the status of systemic hypertension was evaluated separately before the first and second surgeries.

Of the 39 patients included undergoing surgery of one hemisphere, 17 (44%) had preoperative systemic hypertension whereas 22 (56%) did not. At one year following revascularization surgery 16 patients (41%) had systemic hypertension and 30 (59%) did not. As such, only one patient with unilateral disease showed a different status of systemic hypertension following revascularization surgery, in particular, this patient had hypertension prior to revascularization, for which treatment was discontinued one year following surgery (Table 2).

Data were available for 29 patients with bilateral disease. Prior to surgery of the first hemisphere, 16 (55%) had preoperative systemic hypertension and 13 (55%) did not. At one year following surgery of the first hemisphere, overall, two patients had developed systemic hypertension. Consequently, 18 patients (62%) had hypertension. Prior to surgery of the second hemisphere, 17 patients had hypertension (which was longer than one year following surgery of the first hemisphere) and one less had hypertension at one year following surgery.

The median time interval between both surgeries across all patients was 124 days (range 2–603 days), while the majority (83%) was at least after 3 months. Prior to surgery of the second hemisphere, 17 patients (59%) had systemic hypertension and 12 (41%) did not. At one year following revascularization, only one patient demonstrated a change in status with preoperative hypertension being resolved at one year following revascularization of the second hemisphere resulting in 13 patients (45%). This change was also not statistically significant (*p* = 0.656) (Table 2). There were significantly more with ischemic onset disease versus hemorrhagic onset disease in both groups of patients with and without systemic hypertension at one year following surgery (supplementary Table S2).

Patients were divided according to surgery performed on only one hemisphere versus two hemispheres. Only patients for which data were available at all time points pre- and at one year postoperatively were included (39 patients with single hemisphere surgery and 29 with revascularization on both hemispheres). In the case of patients undergoing surgery on two hemispheres, the status of systemic hypertension was evaluated separately before the first and second surgeries. Group comparisons were performed using the McNemar test with *p* < 0.05 considered statistically significant.

### 3.5. Preoperative Hypertension and Occurrence of Adverse Events Following Revascularization Procedures

Next, we asked whether patients with systemic hypertension prior to their first revascularization procedure were more likely to experience perioperative complications compared to those without preoperative hypertension. To this end, we screened patient records for adverse events occurring at any point during patient follow-up. Overall, 18 adverse events were recorded among all patients (10.6% of patients with systemic hypertension and 10.4% of patients without hypertension). Of these, five were repeat revascularizations due to persisting TIAs with bypass occlusion. In two patients, TIAs were reported without correlating bypass occlusion. One patient experienced postoperative aphasia which subsided within five months following surgery and one case of postoperative supplementary motor area syndrome was also observed and resolved before hospital discharge. Four wound-healing disorders and one case of postoperative hemorrhage requiring surgical evacuation were also observed (Table 3).

Adverse events occurring at any point during patient follow-up were included in the analysis. Data are reported as the number of patients in which events occurred.

### 3.6. Influence of Systemic Hypertension in Long-Term Angiographic Development

To examine the possible effects of preoperative systemic hypertension on the long-term development of angioarchitecture in MMA patients following revascularization, analysis of angiographic images acquired at the last clinical follow-up was performed (with a minimum follow-up time of one year following revascularization surgery) for 18 patients undergoing surgery of one hemisphere and 19 patients undergoing surgery for both hemispheres (Table 4).

In patients with preoperative hypertension median follow-up time was 58 months for the first hemisphere treated (range 12–291 months) and 68 months in patients receiving treatment of a second hemisphere (range 12–204 months). In patients without preoperative hypertension, the median follow-up time for the first hemisphere treated was 59 months (range 13–187 months) and in the second hemisphere 78 months (range 13–160 months) (Table 1).

#### 3.6.1. Single Hemisphere

Among patients receiving treatment for one hemisphere, no change was observed in the pattern of leptomeningeal collateralization between those with and without systemic hypertension. Intra-extracranial collateralization remained unchanged in 82% of unilateral cases with systemic hypertension and all those without hypertension. Moyamoya vessels also remained unchanged in 80% of unilateral cases with systemic hypertension and 75% in those without hypertension. Among patients with unilateral surgery, the rate of patients with patent bypasses (grade 1) was lower in patients with systemic hypertension than those without hypertension rates of grade 2 STA-MCA bypass filling were higher in patients with systemic hypertension than those without systemic hypertension. Grade 3 bypass filling was slightly lower in patients with systemic hypertension than in those without systemic hypertension.

#### 3.6.2. Two Hemispheres: First Hemisphere

In all patients with systemic hypertension leptomeningeal collateralization remained unchanged. In patients without hypertension, 25% displayed an increase and 25% a decrease in leptomeningeal collateralization. Regarding extra-intracranial collateralization, more patients with systemic hypertension showed unchanged vascularization patterns (91%) than those without hypertension (71%). Moyamoya vessels also decreased at similar rates among patients with systemic hypertension than those without (18% each). Rates of patients with a patent bypass (grade 1) were higher among patients with systemic hypertension (82%) than those without (71%). Grade 2 STA-MCA bypass filling was slightly more frequent among patients with systemic hypertension (54%) than those without (50%). Grade 3 bypass filling was not found among these patients.

#### 3.6.3. Two Hemispheres: Second Hemisphere

Leptomeningeal collateralization remained unchanged in all patients with and without systemic hypertension. Intra-extracranial collateralization also remained unchanged in all patients with systemic hypertension and 50% of those without hypertension. More patients without systemic hypertension showed a decrease in Moyamoya vessels (88%) than those without hypertension (73%). Bypass patency (grade 1) was present in 91% of patients with systemic hypertension and 63% of patients without systemic hypertension. Patients with systemic hypertension had higher rates of grade 2 STA-MCA bypass filling over time (45%) versus those without (25%).

This analysis was also performed with all patients with a follow-up regardless of the time point (also earlier than one year). Here we found no major deviations from the trends observed in the cohort of follow-up beginning one year after surgery (Supplementary Table S1).

Angiographic images were analyzed as described in the methods section. Results are presented in terms of status at the last follow-up at least one year following the first surgery versus initial preoperative imaging with bypass patency and bypass filling being described at the last follow-up only. Group differences were analyzed using the chi-square test with *p* < 0.05 considered significant.

### 3.7. Systemic Hypertension as a Predictor of Clinical and Radiological Disease Progression in MMA

Furthermore, we hypothesized that systemic hypertension may influence the perfusion in watershed zones in patients with fewer collaterals and/or moyamoya vessels. We therefore further examined whether the presence of systemic hypertension prior to revascularization surgery was associated with impaired angiographic development and/or the occurrence of adverse events. To this end, we analyzed angiographic images at the last follow-up (conducted at least one year postoperatively) after revascularization surgery compared to preoperative images. Analysis was also performed in all follow-up times including those under one year following surgery (Supplementary Table S1). In patients undergoing bilateral revascularization, analysis was performed for each hemisphere separately. Patients undergoing surgery for a second hemisphere were analyzed based on the presence of arterial hypertension before the second surgery.

As we were interested in examining whether preoperative arterial hypertension may have had a negative impact on vascular development and postoperative clinical course, we performed univariate Cox regression analysis to model the relationship of systemic hypertension as an independent variable with events associated with poor angiographic development and clinical outcome at last clinical follow-up. As such, event occurrence was defined as poor (grade 1) STA-MCA bypass filling, the presence of watershed zone anastomoses for leptomeningeal collateralization, extensive transdural collaterals from the middle meningeal artery and abundant moyamoya vessels compared to the preoperative angiography [23]. Bypass occlusion or stenosis as well as poor STA-MCA bypass development were defined compared to the first postoperative angiography. Analysis was performed on all patients with postoperative angiographic imaging at the last clinical follow-up (n = 66 patients). Adverse events included TIAs, stroke, hemorrhage requiring surgical revision, wound healing disorders and supplementary motor syndrome. This analysis was performed on the entire cohort (n = 137 patients).

Using univariate Cox regression analyses, we found no statistically significant predictive role of systemic hypertension in the status of radiological or clinical disease progression. As no variables were found to approach the cut-off of *p* > 0.150, multivariate analysis was not performed (Table 5).

Model summary of univariate Cox regression analysis examining the predictive value of preoperative arterial hypertension on the development of vascular changes associated with disease progression in MMA. Patients undergoing surgery for a second hemisphere were analyzed based on the presence of arterial hypertension before the second surgery. Event occurrence was defined as poor STA-MCA bypass filling, the presence of watershed zone anastomoses for leptomeningeal collateralization, extensive transdural collaterals from the middle meningeal artery and abundant moyamoya vessels compared to the preoperative angiography. Bypass occlusion or stenosis as well as poor STA-MCA bypass development were defined compared to the first postoperative angiography. Adverse events included TIAs, stroke, hemorrhage requiring surgical revision, wound healing disorders, postoperative aphasia and supplementary motor syndrome. Only patients with data available for a minimum of one year following the first surgery were included in the regression analysis.

## 4. Discussion

International population-based studies have found up to 45% of the global adult population to be affected by hypertension [24,25]. The prevalence of systemic hypertension in European, Caucasian populations, similar to the majority of patients examined in this study, has been described to lie between 38–55% [26,27]. Within Germany, the prevalence of systemic hypertension has been reported to lie between 29–41% [28]. When comparing these reports to our cohort of adult patients with MMA we found higher rates of systemic hypertension with 50% of the study population receiving antihypertensive medication prior to revascularization. These findings are in line with the recent reports of pediatric MMA patients who also display higher rates of systemic hypertension [8].

In a recent study by Sutton et al., a significantly higher risk of hypertension (up to 7 times higher than controls) was found and may be due to several factors. These include a difference in population sizes (12 Moyamoya patients in the study from Sutton et al. versus 137 in our analysis and 200 in the study of Kahn et alwhich reports a prevalence of 53% in Moyamoya patients, similar to the 50% found in our analysis). The study of Sutton et al. included only ischemic patients with Moyamoya disease, whereas we included both hemorrhagic and ischemic cases as well as further subforms of the disease (Moyamoya syndrome and unilateral Moyamoya). Finally, the analysis of Sutton et al. was performed in a Hawaiian population, whereas our analysis was conducted in a primarily Caucasian northern European population so the effects of different ethnic backgrounds and their risk for hypertension cannot be entirely ruled out.

Furthermore, it is important to consider the definition of systemic hypertension among MMA patients, for whom disease-specific risk factors such as impaired cerebral hemodynamic control are paramount. Analogous to patients with cardiovascular disease, in which blood pressure reduction below the standard cutoff used to define hypertension has been shown to reduce the risk of cardiovascular events [29], patients with MMA may require specific blood pressure guidelines to adequately reflect disease pathology.

Overall, further studies will be required to define the actual prevalence of disease more clearly in various demographics.

### 4.1. Systemic Hypertension and MMA Severity

In our adult cohort patients with and without systemic hypertension did not significantly differ in age, gender, ethnicity or form of disease onset indicating systemic hypertension in MMA patients may occur independently of other well-known associated factors such as age and gender [30]. Although our analysis explicitly excluded patients with secondary hypertension resulting from primary disease or vascular stenosis elsewhere [9], evidence from pediatric populations has been presented that involvement of the posterior circulation may be associated with the presence of systemic hypertension among children, possibly by triggering a Cushing’s reflex [8]. We found no significant differences in the rate of ischemia on preoperative MRI or impaired CVRC between patients with and without preoperative systemic hypertension. Further analysis including CBF measurements in postoperative patients, which is not routinely performed in our institute if clinical and radiological controls are unremarkable, may provide further insight into possible correlation between regional CBF development and systemic hypertension.

### 4.2. Systemic Hypertension and Angiographic Development

MMA-specific angiographic characteristics such as extensive leptomeningeal and intra-/extracranial collateralization and moyamoya vessels can be considered an adaptive response to chronic cerebral ischemia [23] with extensive leptomeningeal collateralization correlating with advanced disease [31]. Furthermore, adults with fewer leptomeningeal collaterals following indirect revascularization surgery using EDAS were found to be at higher risk for postoperative stroke [19] indicating their importance in the early stages following iatrogenic changes in perfusion patterns via direct or indirect bypass techniques. We therefore next hypothesized that systemic hypertension may help compensate for inadequate perfusion in watershed zones in patients with fewer collaterals and/or moyamoya vessels. Here we found no statistically significant difference between rates of grade 2 bypass filling between patients with and without systemic hypertension so further studies will be necessary to characterize possible influences of blood pressure on bypass patency and angiographic development.

### 4.3. Systemic Hypertension and MMA Progression

Our analysis of systemic hypertension as a predictor for disease progression found no significant influence of the presence of systemic hypertension on the change of either leptomeningeal collaterals, intra-extracranial collaterals, moyamoya vessels or STA-MCA bypass patency between pre- and postoperative angiographic studies. These findings further underscore the hypothesis of a possible protective role of systemic hypertension in the long-term perfusion patterns in adult MMA patients.

The absence of a statistically significant effect between systemic hypertension and patterns of collateralization in MMA patients may be partially explained by the fact that our study population included exclusively adult patients and age-related increases in vascular resistance may act to ameliorate potential effects of systemic hypertension in supporting cerebral perfusion. Furthermore, studies in animal models have proposed that cerebral blood flow through collateral circulation in hypertensive rats is decreased compared to normotensive animals due to their reduced vasodilative capacity [32].

In contrast to pediatric MMA patients in the study of Lee et al., the adult patients in our cohort did not show a significant reduction in the rates of systemic hypertension one year following revascularization surgery. While this may be due to an actual lack of the hypothesized pathophysiological connection between regional cerebrovascular perfusion and systemic blood pressure in MMA patients, following revascularization surgery may also be due to inadequate follow-up time of one year following surgery or the use surrogate parameters to account for hypertension (pharmacological treatment) instead of standardized blood pressure measurements.

Ultimately, the success of revascularization surgery can be measured by the clinical improvement or stabilization of neurological symptoms. In our cohort, there was no significant difference in the occurrence of adverse events (including new or persisting neurological sequelae) in patients with and without systemic hypertension. Despite these findings, the role of intraoperative individualized blood pressure management in MMA patients with compromised cerebral hemodynamics is emerging as a relevant factor in avoiding postoperative ischemia or hyperperfusion [33,34]. This aspect of interdisciplinary hypertension management between Anesthesiology and Neurosurgery certainly warrants further investigation to optimize the intraoperative management of these complex patients.

As insights into the genetic background of MMA continue to expand, several candidate genes linked to abnormal vascular proliferation such as GUCY1A3 or ACTA2 have emerged [35,36]. It remains to be determined if these genetic variations may also affect the presence of systemic hypertension in MMA patients. Further studies are necessary to elucidate possible associations and their clinical relevance.

### 4.4. Limitations

Limitations of the current study include the retrospective, monocentric design in a cohort of 137 patients, of which 68% displayed systemic hypertension. Whereas follow-up times for angiographic analysis in our study varied widely, their overall distribution between the groups of patients with and without systemic hypertension was not statistically significant. Furthermore, the use of antihypertensive medication as a surrogate for standardized blood pressure measurements cannot fully rule out inaccuracies in the categorization of patients as having systemic hypertension or not. Nevertheless, this was an exploratory study to assess the potential role of hypertension in MMA patients without a clear correlation between absolute blood pressure values and effects on blood pressure variability in the course of the disease. Patients with Moyamoya Syndrome may also, as per the definition of the condition, suffer from secondary hypertension due to an associated disease not otherwise included in the exclusion criteria and thereby also possibly falsifying the correct categorization of patients. In future studies, patients with Moyamoya Syndrome may be treated as a separate disease entity to elucidate possible clinically relevant management strategies for these patients.

Based on our findings from the current study, we observed a higher rate of systemic hypertension in patients with MMA; however, this did not translate to significantly higher numbers of clinically or radiologically relevant events for adult patients when compared to MMA patients without hypertension. Nonetheless, based on the nature of impaired cerebral hemodynamics in patients with MMA, further examination of the possible effects of systemic hypertension on perioperative and long-term management of patients is warranted.

## Figures and Tables

**Table 1:** Patient demographics in all patients with and without systemic hypertension prior to first revascularization procedure.

Systemic Hypertension before First Surgery			
Yes		No	*p*
**Total Patients**	68 (50%)	69 (50%)	
Total Hemispheres	104	102	
**Age (years) (median/range)**	44 (19–63)	36 (18–72)	0.179
**Gender**			
M	28 (36%)	14 (20%)	0.425
F	50 (64%)	55 (80%)	
**Ethnicity**			
Caucasion	64 (94%)	65 (94%)	0.475
Asian	3 (4%)	1 (2%)	
Middle Eastern	1 (2%)	3 (4%)	
**Diagnosis**			
MMD	41 (60%)	46 (67%)	0.691
MMS	19 (28%)	15 (21%)	
uMMD	8 (12%)	8 (12%)	
**Onset**			
Ischemic	56 (82%)	58 (84%)	0.941
Hemorrhagic	8 (12%)	7 (10%)	
Other/unknown	4 (6%)	4 (6%)	
**Surgical Strategy (Nr.of Hemispheres)**			
STA/MCA + EDS	47 (69%)	48 (70%)	0.227
STA/MCA + EMS	6 (9%)	4 (6%)	
ECA/MCA	0 (0%)	1 (1%)	
STA–MCA alone	14 (21%)	16 (23%)	
EDGS	1 (1%)	0 (0%)	
**Ischemia on preoperative MRI**	N = 90	N = 94	
Yes	65 (72%)	67 (71%)	0.393
No	25 (27%)	27 (29%)	
**Impaired CVRC**	N = 90	N = 94	
Yes	63 (70%)	73 (78%)	0.824
No	27 (30%)	21 (22%)	
**Suzuki Grade** (Nr. of hemispheres)	N = 93	N = 98	
1	6 (6%)	9 (10%)	0.666
2	29 (32%)	22 (22%)	
3	26 (28%)	29 (30%)	
4	17 (18%)	24 (24%)	
5	13 (14%)	12 (12%)	
6	2 (2%)	2 (2%)	
**Berlin Grade** (Nr. of hemispheres)	N = 90	N = 94	
1	16 (18%)	13 (14%)	0.393
2	38 (42%)	46 (49%)	
3	36 (40%)	35 (37%)	
**Last Follow–up for angiographic analysis** (median/range in months)			
First Hemisphere	55 (3–291)	58 (6–187)	0.849
Second Hemisphere	42 (1–204)	52 (11–160)	

**Table 2:** Development of arterial hypertension following revascularization procedures.

	Systemic Hypertension			
	Yes		No	*p*
**Single Hemisphere** **n = 39**	Preoperative	17 (44%)	22 (56%)	0.500
	1 Year Postoperative	16 (41%)	23 (59%)	
**Two Hemispheres** **n = 29**	**First Hemisphere**			
	Preoperative	16 (55%)	13 (45%)	0.688
	1 Year Postoperative	18 (62%)	11 (38%)	
	**Second Hemisphere**			
	Preoperative	17 (59%)	12 (41%)	0.656
	1 Year Postoperative	16 (55%)	13 (45%)	

**Table 3:** Preoperative hypertension and occurrence of adverse events following revascularization procedures.

Systemic Hypertension Before First Surgery			
	Yes	No	
Repeat Revascularization	2	3	
TIAs	0	3	
Ischemic Stroke	3	1	
Hemorrhage	0	1	
SMA Syndrome	0	1	
Wound healing disorder	3	1	
**Total adverse events**	**8 (10.6%)**	**10 (10.4%)**	***p* = 0.632**

**Table 4:** Angiographic Development.

Systemic Hypertension					
			Yes	No	*p*
**Single Hemisphere** **n = 18**		**Leptomeningeal Collateralization**			
		Unchanged	10 (100%)	8 (100%)	0.493
		Increased	0	0	
		Decreased	0	0	
		**Extra-Intracranial Collateralization**			
		Unchanged	9 (82%)	7 (100%)	0.500
		Increased	1 (9%)	0	
		Decreased	1 (9%)	0	
		**Moyamoya Vessels**			
		Unchanged	8 (80%)	6 (75%)	0.309
		Decreased	2 (20%)	2 (25%)	
		**Bypass Patency**			
		Patent	7 (70%)	6 (75%)	0.497
		Stenosed/Occluded	3 (30%)	2 (25%)	
		**STA-MCA Bypass Filling**			
		Grade 1	5 (50%)	4 (50%)	0.542
		Grade 2	4 (40%)	3 (38%)	
		Grade 3	1 (10%)	1 (12%)	
**Two Hemispheres** **n = 19**	**First Hemisphere**	**Leptomeningeal Collateralization**			
		Unchanged	11 (100%)	4 (50%)	0.343
		Increased	0	2 (25%	
		Decreased	0	2 (25%)	
		**Extra-Intracranial Collateralization**			
		Unchanged	10 (91%)	5 (71%)	0.786
		Increased	0	1 (11%)	
		Decreased	1 (9%)	2 (18%)	
		**Moyamoya Vessels**			
		Unchanged	9 (82%)	7 (82%)	0.335
		Decreased	2 (18%)	1 (18%)	
		**Bypass Patency**			
		Patent	9 (82%)	5 (71%)	0.523
		Stenosed/Occluded	2 (18%)	3 (29%)	
		**STA-MCA Bypass Filling**			
		Grade 1	5 (45%)	4 (50%)	0.675
		Grade 2	6 (54%)	4 (50%)	
		Grade 3	0	0	
	**Second Hemisphere**	**Leptomeningeal Collateralization**			
		Unchanged	11 (100%)	6 (75%)	0.493
		Increased	0	0	
		Decreased	0	2 (25%)	
		**Extra-Intracranial Collateralization**			
		Unchanged	11 (100%)	4 (50%)	0.383
		Increased	0	2 (25%)	
		Decreased	0	2 (25%)	
		**Moyamoya Vessels**			
		Unchanged	8 (73%)	7 (88%)	0.604
		Decreased	3 (27%)	1 (12%)	
		Bypass Patency			
		Patent	10 (91%)	5 (63%)	0.583
		Stenosed/Occluded	1 (9%)	3 (37%)	
		**STA-MCA Bypass Filling**			
		Grade 1	6 (54%)	5 (63%)	0.562
		Grade 2	5 (45%)	2 (25%)	
		Grade 3	0	1 (12%)	

**Table 5:** Arterial hypertension as a predictor of angiographic characteristics and adverse events in MMA.

**First Hemisphere**			
	**Hazard Ratio**	**CI (95%)**	***p***
**Leptomeningeal Collateralization**	1.536	0.139–17.025	0.727
**Extra-Intracranial Collateralization**	0.308	0.032–3.001	0.310
**Moyamoya Vessels**	0.991	0.356–2.760	0.986
**Bypass Patency**	0.670	0.310–1.451	0.310
**STA-MCA Bypass Filling**	1.530	0.726–3.226	0.254
**Adverse Event**	0.764	0.107–5.435	0.788
**Second Hemisphere**			
	**Hazard Ratio**	**CI (95%)**	***p***
**Leptomeningeal Collateralization**	1.642	0.231–11.688	0.621
**Extra-Intracranial Collateralization**	1.913	0.150–24.489	0.618
**Moyamoya Vessels**	0.908	0.363–2.273	0.836
**Bypass Patency**	2.240	0.518–9.681	0.280
**STA-MCA Bypass Filling**	1.007	0.333–3.043	0.990
**Adverse Event**	0.712	0.064–7.893	0.782

## Data Availability

Data is available upon reasonable written request.

## Supplementary Materials

The following supporting information can be downloaded at: https://www.mdpi.com/article/10.3390/jcm12134219/s1, Supplementary Table S1: Angiographic Development over all time periods. Supplementary Table S2: Patients with systemic hypertension at one year following surgery.

## References

[1] Harper A.M. (1966). Autoregulation of cerebral blood flow: Influence of the arterial blood pressure on the blood flow through the cerebral cortex. J. Neurol. Neurosurg. Psychiatry.

[2] Feldmann E., Skolnick B.E. (1999). Cerebral hemodynamics, autoregulation, and blood pressure management. J. Stroke Cerebrovasc. Dis..

[3] Suzuki J., Takaku A. (1969). Cerebrovascular “moyamoya” disease. Disease showing abnormal net-like vessels in base of brain. Arch. Neurol..

[4] Research Committee on the Pathology and Treatment of Spontaneous Occlusion of the Circle of Willis, Health Labour Sciences Research Grant for Research on Measures for Infractable Diseases (2012). Guidelines for diagnosis and treatment of moyamoya disease (spontaneous occlusion of the circle of Willis). Neurol. Med. Chir..

[5] Acker G., Goerdes S., Schneider U.C., Schmiedek P., Czabanka M., Vajkoczy P. (2015). Distinct clinical and radiographic characteristics of moyamoya disease amongst European Caucasians. Eur. J. Neurol..

[6] Jeon J.P., Kim J.E., Cho W.-S., Bang J.S., Son Y.-J., Oh C.W. (2018). Meta-analysis of the surgical outcomes of symptomatic moyamoya disease in adults. J. Neurosurg..

[7] Kuroda S., Hashimoto N., Yoshimoto T., Iwasaki Y. (2007). Radiological findings, clinical course, and outcome in asymptomatic moyamoya disease: Results of multicenter survey in Japan. Stroke.

[8] Lee J., Kim S.K., Kang H.G., Ha I.S., Wang K.C., Lee J.Y., Phi J.H. (2019). High prevalence of systemic hypertension in pediatric patients with moyamoya disease years after surgical treatment. J. Neurosurg. Pediatr..

[9] Yamada I., Himeno Y., Matsushima Y., Shibuya H. (2000). Renal artery lesions in patients with moyamoya disease: Angiographic findings. Stroke.

[10] Bang O.Y., Fujimura M., Kim S.K. (2016). The Pathophysiology of Moyamoya Disease: An Update. J. Stroke.

[11] Sutton C.X.Y., Carrazana E., Mitchell C., Viereck J., Liow K.K., Ghaffari-Rafi A. (2022). Identification of associations and distinguishing moyamoya disease from ischemic strokes of other etiologies: A retrospective case-control study. Ann. Med. Surg..

[12] Kahn A., Kaur G., Stein L., Tuhrim S., Dhamoon M.S. (2018). Treatment course and outcomes after revascularization surgery for moyamoya disease in adults. J. Neurol..

[13] Williams B., Mancia G., Spiering W., Agabiti Rosei E., Azizi M., Burnier M., Clement D.L., Coca A., De Simone G., Dominiczak A. (2018). 2018 ESC/ESH Guidelines for the management of arterial hypertension: The Task Force for the management of arterial hypertension of the European Society of Cardiology and the European Society of Hypertension: The Task Force for the management of arterial hypertension of the European Society of Cardiology and the European Society of Hypertension. J. Hypertens..

[14] Hart E.C. (2016). Human hypertension, sympathetic activity and the selfish brain. Exp. Physiol..

[15] Czabanka M., Peña-Tapia P., Schubert G., Heppner F., Martus P., Horn P., Schmiedek P., Vajkoczy P. (2011). Proposal for a new grading of Moyamoya disease in adult patients. Cerebrovasc. Dis..

[16] Acker G., Giampiccolo D., Rubarth K., Mertens R., Zdunczyk A., Hardt J., Jussen D., Schneider H., Rosenstock T., Mueller V. (2021). Motor excitability in bilateral moyamoya vasculopathy and the impact of revascularization. Neurosurg. Focus.

[17] Lucia K., Acker G., Mrosk F., Beyaztas D., Vajkoczy P. (2022). Longitudinal angiographic characterization of the efficacy of combined cerebral revascularization using minimally invasive encephalodurosynangiosis in patients with moyamoya angiopathy. Neurosurg. Rev..

[18] Czabanka M., Peña-Tapia P., Scharf J., Schubert G., Münch E., Horn P., Schmiedek P., Vajkoczy P. (2011). Characterization of direct and indirect cerebral revascularization for the treatment of European patients with moyamoya disease. Cerebrovasc. Dis..

[19] Liu Z., Han C., Wang H., Zhang Q., Li S., Bao X., Zhang Z., Duan L. (2020). Clinical characteristics and leptomeningeal collateral status in pediatric and adult patients with ischemic moyamoya disease. CNS Neurosci. Ther..

[20] Czabanka M., Acker G., Jussen D., Finger T., Pena-Tapia P., Schubert G.A., Scharf J., Martus P., Schmiedek P., Vajkoczy P. (2014). Collateralization and ischemia in hemodynamic cerebrovascular insufficiency. Acta Neurochir..

[21] Storey A., Scott R.M., Robertson R., Smith E. (2017). Preoperative transdural collateral vessels in moyamoya as radiographic biomarkers of disease. J. Neurosurg. Pediatr..

[22] Zhao Y., Li J., Lu J., Zhang Q., Zhang D., Wang R., Zhao Y., Chen X. (2019). Predictors of neoangiogenesis after indirect revascularization in moyamoya disease: A multicenter retrospective study. J. Neurosurg..

[23] Liebeskind D.S. (2003). Collateral circulation. Stroke.

[24] Zhou B., Bentham J., Di Cesare M., Bixby H., Danaei G., Cowan M.J., Paciorek C.J., Singh G., Hajifathalian K., Bennett J.E. (2017). Worldwide trends in blood pressure from 1975 to 2015: A pooled analysis of 1479 population-based measurement studies with 19·1 million participants. Lancet.

[25] Mills K.T., Bundy J.D., Kelly T.N., Reed J., Kearney P.M., Reynolds K., Chen J., He J. (2016). Global Disparities of Hypertension Prevalence and Control: A Systematic Analysis of Population-Based Studies From 90 Countries. Circulation.

[26] Wolf-Maier K., Cooper R.S., Banegas J.R., Giampaoli S., Hense H.W., Joffres M., Kastarinen M., Poulter N., Primatesta P., Rodríguez-Artalejo F. (2003). Hypertension prevalence and blood pressure levels in 6 European countries, Canada, and the United States. JAMA.

[27] Wolf-Maier K., Cooper R.S., Kramer H., Banegas J.R., Giampaoli S., Joffres M.R., Poulter N., Primatesta P., Stegmayr B., Thamm M. (2004). Hypertension treatment and control in five European countries, Canada, and the United States. Hypertension.

[28] Meisinger C., Heier M., Völzke H., Löwel H., Mitusch R., Hense H.-W., Lüdemann J. (2006). Regional disparities of hypertension prevalence and management within Germany. J. Hypertens..

[29] Rahimi K., Bidel Z., Nazarzadeh M., Copland E., Canoy D., Ramakrishnan R., Pinho-Gomes A.C., Woodward M., Adler A., Agodoa L. (2021). Pharmacological Blood Pressure Lowering Treatment Trialists’ Collaboration, Pharmacological blood pressure lowering for primary and secondary prevention of cardiovascular disease across different levels of blood pressure: An individual participant-level data meta-analysis. Lancet.

[30] Gillis E.E., Sullivan J.C. (2016). Sex Differences in Hypertension: Recent Advances. Hypertension.

[31] Strother M., Anderson M., Singer R., Du L., Moore R., Shyr Y., Ladner T., Arteaga D., Day M., Clemmons P. (2014). Cerebrovascular collaterals correlate with disease severity in adult North American patients with Moyamoya disease. AJNR Am. J. Neuroradiol..

[32] Coyle P., Heistad D.D. (1986). Blood flow through cerebral collateral vessels in hypertensive and normotensive rats. Hypertension.

[33] Li J., Zhao Y., Zhao M., Cao P., Liu X., Ren H., Zhang D., Zhang Y., Wang R., Zhao J. (2020). High variance of intraoperative blood pressure predicts early cerebral infarction after revascularization surgery in patients with Moyamoya disease. Neurosurg. Rev..

[34] Li C., Zhang N., Yu S., Xu Y., Yao Y., Zeng M., Li D., Xia C. (2021). Individualized Perioperative Blood Pressure Management for Adult Moyamoya Disease: Experience from 186 Consecutive Procedures. J. Stroke Cerebrovasc. Dis..

[35] Hervé D., Philippi A., Belbouab R., Zerah M., Chabrier S., Collardeau-Frachon S., Bergametti F., Essongue A., Berrou E., Krivosic V. (2014). Loss of α1β1 soluble guanylate cyclase, the major nitric oxide receptor, leads to moyamoya and achalasia. Am. J. Hum. Genet..

[36] Guo D.-C., Papke C.L., Tran-Fadulu V., Regalado E.S., Avidan N., Johnson R.J., Kim D.H., Pannu H., Willing M.C., Sparks E. (2009). Mutations in smooth muscle alpha-actin (ACTA2) cause coronary artery disease, stroke, and Moyamoya disease, along with thoracic aortic disease. Am. J. Hum. Genet..

