# Supplementary material for: The Development and Effect of Systemic Hypertension on Clinical and Radiological Outcome in Adult Moyamoya Angiopathy Following Revascularization Surgery: Experience of a Single European Institution
Source: J Clin Med. 2023 Jun 23;12(13):4219. doi: 10.3390/jcm12134219 (PMC10342858; doi:10.3390/jcm12134219)
Supplement: Supplementary file 1 [file jcm-12-04219-s001.zip › jcm-2376798-supplementary.pdf]

Table S1:

Patients with systemic hypertension at one year following surgery			
	**Ischemic Onset**	**Hemorrhagic Onset**	**p**
**Yes (n = 32)**	28	4	***0.010***
**No (n = 36)**	31	5	***0.003***

Results are given for all patients including both uni- and bilateral disease. For patients with bilateral disease results are drawn from status after revascularization of the second hemisphere. Group comparisons were performed using the McNemar test with p<0.05 considered statistically significant.

Table S2:

Patients with systemic hypertension at one year following surgery			
	**Ischemic Onset**	**Hemorrhagic Onset**	**p**
**Yes (n=32)**	28	4	***0.010***
**No (n=36)**	31	5	***0.003***

Results are given for all patients including both uni- and bilateral disease. For patients with bilateral disease results are drawn from status after revascularization of the second hemisphere. Group comparison were performed using the McNemar test with p<0.05 considered statistically significant.
